# Mind the Rhythm: ECG QT Dispersion and Cognition in Healthy Older Adults

**DOI:** 10.3389/fpsyg.2020.566341

**Published:** 2020-09-30

**Authors:** Tudor Vrinceanu, Geneviève Lagacé-Lavoie, Navin Kaushal, Alida Esmail, T. T. Minh Vu, Nicolas Berryman, Anil Nigam, Louis Bherer

**Affiliations:** ^1^Department of Medicine, Université de Montréal, Montreal, QC, Canada; ^2^Research Centre, Montreal Heart Institute, Montreal, QC, Canada; ^3^Research Centre, Institut Universitaire de Gériatrie de Montréal, Montreal, QC, Canada; ^4^Centre Hospitalier de l’Université de Montréal, Montreal, QC, Canada; ^5^Department of Health Sciences, School of Health & Human Sciences, Indiana University, Indianapolis, IN, United States; ^6^École de Réadaptation, Faculté de Médecine, Université de Montréal, Montreal, QC, Canada; ^7^Centre de Recherché du CHUM, Montreal, QC, Canada; ^8^Département des Sciences de l’Activité Physique, Université du Québec à Montréal, Montreal, QC, Canada

**Keywords:** cognition, ventricular repolarization dispersion, aging, cardiovascular health, autonomic function

## Abstract

**Background:**

Autonomic function has been linked to cognitive abilities in aging. Even in non-clinical states, a certain variability in heart rhythm regulation can be measured with QT dispersion (QTcD), an ECG marker of ventricular repolarization which has been linked to autonomic function and cardiovascular health. QTcD has been shown to be higher in individuals with mild cognitive impairment, and the highest in individuals with Alzheimer’s disease. The goal of this study was to see if QTcD is associated with cognitive performance in healthy individuals.

**Methods:**

Sixty-three healthy inactive older adults (> 60 years) completed an extensive cognitive assessment (including inhibition, divided attention, updating, working memory, and processing speed), a physical fitness assessment, and underwent a resting ECG.

**Results:**

After controlling for age, sex, and education, QTcD significantly predicted global cognition (MoCA) scores (*R*^2^ = 0.17, *F*_(__4__.__58__)_ = 3.00, *p* < 0.03, β = −0.36). Exploratory analysis on the MoCA subcomponents revealed a significant association between the visual/executive subcomponent and QTcD (*R*^2^ = 0.12, *F*_(1__.6__1)_ = 7.99, *p* < 0.01, β = −0.34). In individuals with high QTcD, QTcD values were linked to executive functions (*R*^2^ = 0.37), processing speed (*R*^2^ = 0.34), and dual-task performances (*R*^2^ = 0.47). No significant associations were found within the low QTcD group.

**Conclusion:**

This study shows an association between ventricular repolarization (QTcD) and cognitive performance, in particular speed and executive functions, in healthy older adults. The results provide further support for linking autonomic heart regulation and age-related cognitive changes, and suggest that deviations on ECG, even within-normal range, could help detect early cognitive deficits.

## Introduction

Studies have shown an association between variability of specific cardiac ECG parameters and cognition in older adults (Frewen et al., 2013), suggesting a link between autonomic regulation and brain health (Obara et al., 2018). This relationship is thought to be even more evident in individuals suffering from cardiovascular or neurodegenerative diseases, further emphasizing a potential bidirectional link between heart and brain (Toledo and Junqueira, 2010; Keary et al., 2012). Few recent studies have suggested ventricular repolarization ECG measures as one potential marker linking autonomic cardiovascular regulation and cognition (Lucas et al., 2010; Mahinrad et al., 2019). A better understanding of the association between specific ECG parameters and different cognitive functions would allow a better characterization of the heart-brain continuum and help identify better indices or proxies of early subtle cognitive decline. This study investigated whether QTcD, a measure of ventricular repolarization reflecting autonomic cardiovascular regulation, is related to cognitive performance in healthy inactive older adults.

The autonomic nervous system (ANS) comprised of two independent branches, sympathetic and parasympathetic, is thought to have a direct impact on the heart through the vagus nerve (parasympathetic influence). With increased age, most studies have reported a loss of the balance between the sympathetic and parasympathetic tone (Abhishekh et al., 2013). More precisely, it has been shown that advanced age is marked by a gradual loss of the vagal tone, and a relative increase in sympathetic activity (Abhishekh et al., 2013). Those changes are multidetermined often involving a gradual degradation of neuronal structures responsible for autonomic control, and a decrease in the body’s sensitivity to ANS triggers (Hotta and Uchida, 2010). Those age-related changes in autonomic function have also been associated with other lifestyle risk factors (such as lack of physical activity, poor diet, smoking), with other age related health deteriorations (higher blood pressure, dyslipidemia, cognitive decline), and it has been shown to even predict the incidence of cardiovascular disease in longitudinal studies (Liao et al., 1996, 1997; Forte et al., 2019).

Most of the studies investigating the link between the ANS and cognition have used heart rate variability (HRV) as the primary proxy to asses autonomic regulation. This method takes advantage of the autonomic control of the inter beat variability by measuring RR intervals over a longer period of time (usually over a few minutes). Analyses conducted in the time domain, frequency domain, or using non-linear analyses of the averaged inter-beat intervals can be used to extract specific values thought to reflect sympathetic or parasympathetic activity (Forte et al., 2019).

Poor autonomic regulation of the heart has been associated with neurovascular damages and cognitive decline. Specifically, reduced HRV in older adults has been shown to predict lower global cognitive performance beyond the expected impact of cardiovascular risk factors or other comorbidities (Zeki Al Hazzouri et al., 2014; Mahinrad et al., 2016), and it has been suggested as an early biomarker able to detect future cognitive decline (Forte et al., 2019). In addition, autonomic dysregulation (measured through a multitude of methodologies) has been found to correlate with neurodegeneration and deterioration of brain functional activity among healthy individuals and those with dementia (Allan et al., 2007; Lin et al., 2017; Obara et al., 2018). However, due to the large quantity of data required to be recorded and analyzed, the HRV is extracted from a digital ECG recording and the scores are calculated using automated software. This makes it harder to implement in the routine clinical setting.

Some studies investigating the link between autonomic cardiac control and cognition have also used other cardiac markers beyond HRV like ventricular repolarization (QT interval on the ECG). The autonomic tone is known to impact the rate of cardiac repolarization and the difference between the longest and the shortest QT intervals (QT dispersion) is thought to reflect the heterogeneity in cardiac repolarization, with higher values being linked with an impaired ANS functioning and other cardiovascular problems (Ahnve and Vallin, 1982; Rosen et al., 1992; Malik and Batchvarov, 2000; Guntekin et al., 2011; Monitillo et al., 2016). The advantage of this marker is that it can be easily extracted from a routine paper ECG without requiring any other software for analysis. Studies have previously linked ventricular repolarization to cognitive functioning, however, most of the studies have focused on individuals with cognitive deficiencies or with cardiovascular problems. For example, high heterogeneity in ventricular depolarization (spatial QRS-T angle) was linked to steeper decline in processing speed, and immediate and delayed recall after a 3.2 years follow-up in individuals with cardiovascular disease or at high cardiovascular risk (Mahinrad et al., 2019). High heterogeneity in ventricular repolarization (QTcD) has also been shown to be higher in older individuals with clinical cognitive decline than in healthy individuals (Zulli et al., 2005; Coppola et al., 2013). It is worth noting that this relationship was dependent on the severity of the cognitive decline, that is, individuals with Alzheimer’s disease (AD) had higher QTcD values than individuals with mild cognitive impairment (MCI). Whether high QTcD is linked to cognition in healthy asymptomatic individuals has never been explored. Moreover, it is not clear if certain cognitive functions are more likely to be associated with ventricular repolarization markers since the previous studies used clinical global cognitive tests.

Like all other markers of autonomic cardiac control, QTcD is linked to cardiovascular health (Monitillo et al., 2016). As a result, the present study included only healthy individuals without any cardiovascular disease. This would allow for the investigation of the link between QTcD and cognition independent of cardiovascular disease. Due to methodological challenges in fully understanding the mechanisms captured by QTcD, it is not yet recommend its use in the clinical setting, however, the investigation of QTcD in the experimental settings has been recommended in order to better understand its significance, and its ability to detect individuals at risk (Malik et al., 2000; Rautaharju et al., 2009). It has also been suggested that QTcD values might reflect measurement error within normal range (Malik and Batchvarov, 2000). However, confidence in its interpretation is increased within higher values as this might reflect unusual activity beyond measurement error (Malik and Batchvarov, 2000). Since there are no current recommendations for QTcD cutoff values in healthy individuals, the present study will use a median split on the QTcD value in order to identify if participants that have higher QTcD values might show a stronger association with the cognitive variables measured.

Finally, the variability in autonomic regulation of cardiovascular function has also been associated with physical fitness, which is also known to be linked to cognitive performance in older adults (Bjornstad et al., 1991, 1993a; Baur et al., 2012; Bherer et al., 2019). It is generally accepted that higher fit individuals have a lower resting heart rate, better autonomic regulation, and better cognitive functions (Bjornstad et al., 1993b; Dupuy et al., 2018). Therefore, the cardiovascular fitness level of the participants must be taken into account as a potential mediator when investigating the link between autonomic regulation of cardiovascular function and cognition. The present study sought to investigate whether higher QTcD would predict poorer cognitive performance in healthy inactive asymptomatic older adults while considering fitness level as a potential mediator.

## Materials and Methods

The cross-sectional data used for the current study was part of a larger registered physical activity intervention clinical trial (clinicaltrials.gov identifier: NCT02455258) comparing the impact of a 3-months aerobic training, dance movement training and a wait-list control group on cognition and quality of life (Vrinceanu et al., 2019; Esmail et al., 2020). For a more detailed description of the procedure, and an in-depth breakdown of the composite scores used please see Esmail et al. (2020). In order to test the hypothesis of this paper the pre-intervention data from all participants was used. The study has been approved by the ethics board of the research institution, and all participants offered their informed consent before starting the study.

### Participants

Sixty-three healthy, inactive, older adults over the age of 60 (*M* = 67.48, range = 60–86) recruited from the community agreed to participate in the study (Table 1). Exclusion criteria were: cognitive impairment [Mini-Mental State Examination (MMSE) score ≤ 24], engagement in an exercise program (of 150 min/week or more) in the last year, impaired mobility, any surgery involving general anesthesia in the past year, diagnosis of any orthopedic, neurological, cardiovascular, respiratory, progressive neurologic, and somatic disease in the past 6 months, history of smoking in the past 5 years, drinking more than two standard drinks per day. Based on a geriatric assessment, the sample used did not suffer from any condition known to interact with the ECG QT (e.g., heart disease, diabetes, central nervous system disease, uncontrolled thyroid disease, folic acid intake, B12 deficiency). None of the participants were taking any QT-related drugs. Participants were also excluded if they were taking any other medication that could impact the variables of interest of the RCT (related to cognitive function, or ability to exercise). In the case in which they were taking any cardiovascular medication, they were included in the study if the doctor considered it did not have an interaction with the variables of interest of this paper (cognitive ability, and cardiac electrical activity) and if there was no major change in their medication in the past 6 months. This information has been assessed by the medical doctor at two points, once at the inclusion visit, and once when the data was compiled for analysis in the preparation of this paper. During the medical visit the geriatrician evaluated the presence of any of the conditions mentioned above and assessed the overall health status of the participants. Participants were considered healthy for their age if they didn’t present any of the conditions listed.

**TABLE 1:** Baseline descriptive data.

**Characteristic**	**Mean (SD)**		
	**Full sample (*n* = 63)**	**High QTcD (*n* = 29)**	**Low QTcD (*n* = 34)**
Age	67.48 (5.37)	67.75 (5.65)	67.26 (5.20)
BMI (kg/m^2^)*	26.93 (4.96)	25.39 (4.88)	28.16 (4.74)
Education Level (years)	14.94 (3.40)	14.95 (2.72)	14.94 (3.90)
Women %*	77.8%	89.3%	68.6%
GDS	4.77 (5.56)	6.30 (6.57)	3.52 (4.30)
MMSE	28.08 (1.41)	28.25 (1.14)	27.94 (1.59)
MoCA	26.62 (2.42)	26.39 (2.26)	26.80 (2.55)
VO_2_Peak (ml.kg^–1^.min^–1^)	21.55 (5.25)	21.95 (5.38)	21.22 (5.19)
10 mW (m/s)	1.81 (0.26)	1.84 (0.23)	1.77 (0.27)
QT (msec)	394.05 (25.64)	400.21 (27.37)	388.97 (23.30)
QTcD (msec)*	52.76 (17.63)	69.14 (12.15)	39.66 (7.19)
PR (msec)	162.38 (23.66)	165.07 (20.39)	160.23 (26.09)
QRS (msec)	83.11 (7.35)	82.21 (7.97)	83.83 (6.85)
RR (sec)	0.93 (0.16)	0.94 (0.16)	0.92 (0.16)
HR	66.68 (11.42)	66.18 (12.81)	67.09 (10.35)
SBP	137.73 (18.17)	134.56 (20.31)	140.41 (16.0)
DBP	79.29 (9.46)	78.41 (10.82)	80.03 (8.24)
Cardio. Comorbidities	0.62 (0.86)	0.46 (0.66)	0.71 (0.95)

BMI, Body Mass Index; GDS, Geriatric Depression Scale; MMSE, Mini-Mental State Examination; MoCA, Montreal Cognitive Assessment; 10 mW,10 meters walk test speed; QTcD, QT dispersion–controlled for heart rate; HR, resting heart rate; SBP, Systolic Blood Pressure; DBP, Diastolic Blood Pressure; Cardio. Comorbidities, sum of cardiovascular comorbidities based on the presence of: hypertension, diabetes, dyslipidemia, and stable ischemic heart disease. *p < 0.05.

### Procedure

Participants completed all assessments over the course of 3 days. After the medical exam, participants underwent an extensive neuropsychological assessment, a 10-meter walk test, and a VO_2_Peak to assess their mobility and physical fitness level. A resting state ECG measure was taken prior to the VO_2_Peak test.

### Assessments

#### ECG

A standard medical 12-lead seated resting ECG was collected for all participants for 5 min using Schiller Cardiovit AT-10 Plus. A trained medical doctor who did not participate in testing analyzed the paper-based ECG data and extracted manually the RR, PR, QRS, and QT according to standard medical procedure. The last 10-s paper strip (or the last 12 QRS complexes if fewer were present in the last 10 s) was used and the measurements were made visually, using a ruler with the smallest unit being 0.5 mm corresponding to 20 ms. The QT intervals were measured according to standard criteria, from the start of QRS complex to the end of the T wave. The end of the wave was defined as the point of return to the isoelectric line. If a U wave was present, the QT interval was measured to the nadir of the curve between the T and the U waves. In each participant, at least nine leads had to be clearly visible. From the QT interval the QTc was calculated using the Bazett’s formula to control for heart rate (Bazett, 1920). QTc dispersion (QTcD) values were calculated by subtracting from the highest QTc value the lowest one (Zulli et al., 2005).

#### Cognitive Assessments

A detailed description of all cognitive variables and the detailed breakdown of the cognitive composite scores can be found in Esmail et al. (2020). All cognitive assessments were done by a neuropsychologist, or a trained psychology student. Global cognition was measured using the Montreal Cognitive Assessment (MoCA) (Nasreddine et al., 2005). The total score on the MoCA (out of 30) is obtained by adding up all the scores obtained in each subsection of the MoCA: visuospatial/executive, naming, memory, attention, language, abstraction, delayed recall, and orientation. Most of the MoCA subscales are scored out of 1, 2 or 3 points, except visuospatial/executive and delayed recall being scored out of 5, and orientation being scored out of six points. The results using the MoCA subscales should be interpreted with caution due to its restricted range and limited validity. The Digit Symbol Substitution Test was used to assess processing speed (Jaeger, 2018).

Cognitive functions were further measured using three tablet tasks: N-back [adapted from Owen et al. (2005)], Stroop [adapted from Sedo (2004)]; and a divided attention Dual Task [adapted from Lussier et al. (2017)], during which reaction time and accuracy were recorded (Esmail et al., 2020). During the N-back task participants had to identify if the digit present on the screen is the same or different than the digit presented N-positions before, with N gradually increasing from 1 to 2. The Digit Stroop task used was modified for tablet and it included four different conditions: reading, counting, inhibition, and switching. In the inhibition block, the participant had to identify the number of identical digits present on the screen, while inhibiting the automated action of reading the digits. For the switching block, participants were instructed to either name the number of items (inhibition trials) or, if the items were surrounded by a white border, to provide the value of the digits (reading trials). In the Dual-Task (DT), participants were asked to perform two visual discrimination tasks, alone or concurrently. The task had three conditions: Single Pure (SP), Single Mixed (SM), and Dual Mixed (DM). In the SP condition participants had to identify which one of three symbols (planets for one task and animals for the other task) was presented on the screen. In the SM condition, participants had to identify one stimulus among any of the stimuli of the two tasks. In the DM condition, participants had to identify two stimuli (one from each of the sets) presented at the same time. In addition to response speed and accuracy, two types of costs were also extracted from the DT (Esmail et al., 2020). Dual task cost was calculated by subtracting the RT during the SM condition from the RT in the DM condition. This cost reflects the ability to direct attention and answer to two different stimuli. The task set cost was calculating by subtracting the RT in the SP condition from the RT in the SM condition. This cost reflects the ability to prepare and maintain attention to all potential stimuli.

Two composite scores were created with the data obtained from the tablet tasks. An executive cognitive composite score was comprised of (1) 2-back accuracy of the N-back task, (2) average SM trials’ RT from the DT, (3) average DM trials’ RT from the DT, (4) average RT from the Stroop inhibition block, and (5) average RT from the Stroop switching block. A second non-executive cognitive composite score was comprised of (1) 1-back accuracy of the N-back, (2) average SP trials’ RT from the DT, and (3) average RT from the Stroop reading block. All components within each composite score correlated with each other, and they showed a convincing degree of internal consistency (Cronbach’s α = 0.83 for the executive score, and α = 70 for the non-executive score).

#### Physical Assessments

The 10-meter walk test was used to assess participants’ mobility. Starting from a standstill position, participants had to walk as fast as they could in a straight line without running over 10 meters. Time was recorded.

VO_2_Peak was assessed during a maximal continuous graded test performed on a stationary bicycle (Corival Recumbent, Lode BV, Groningen, Netherlands). For more details on the test please see Berryman et al. (2013). Initial mechanical power was set at 50 W for males and 35 W for females. Power was then increased by 15 W every 60 s, with a fixed pedaling cadence of 60 to 80 rpm. Termination criterion was the inability to maintain the required pedaling cadence. The highest VO_2_Peak over a 30-s period during the test was considered as VO_2_Peak (in ml.kg^–1^.min^–1^).

### Statistical Analysis

The study’s objectives were investigated using IBM SPSS version 24.0 for Windows (IBM, Inc., Chicago, IL, United States). In order to decrease the impact of outliers, data were winsorized at 3 SD away from the mean. Normality of data distribution was determined by following recommendations by Tabachnick and Fidell (2019) which included assessing the kurtosis and skewness of all variables. Prior to conducting any analyses, the sample was partitioned and binary coded as high (QTcD > 47 ms) vs. low (QTcD ≤ 47 ms) based on a median split. This has been considered due to the high range of QTcD values observed in the present sample (range: 20–105 ms). Zero-order bivariate Pearson correlations were performed between the QTcD values and the cognitive variables before testing the hypotheses, both in the high QTcD and low QTcD subgroups and on the two subgroups combined (see Table 2). This was followed by a series of multiple linear regressions on the significantly correlated relationships to test the study’s hypotheses. All regression analyses controlled for the effect of age, sex, and education, due to their documented impact on cognitive aging.

**TABLE 2:** Pearson correlation coefficients between QTcD and the cognitive variables.

	**MoCA**	**Exec.**	**Non-Exec.**	**DSST**	**TSC**	**DTC**	**MoCA V-E.**	**MoCA Naming**	**MoCA Attention**	**MoCA Language**	**MoCA Abstr.**	**MoCA Mem.**	**MoCA Orien.**
QTcD^a^	−0.28*	−0.04	−0.03	−0.04	0.20	−0.03	−0.34**	−0.24	−0.21	−0.13	.01	0.02	0.11
QTcD^b^	−0.58**	−0.40*	−0.25	−0.41*	0.42*	−0.28	−0.39*	−0.17	−0.05	−0.62**	−0.44*	−0.24	/
QTcD^c^	−0.19	−0.14	−0.13	−0.17	0.13	−0.07	−0.15	0.22	−0.06	−0.14	0.17	−0.08	−0.15

MoCA, Montreal Cognitive Assessment; Exec., executive composite score; Non-Exec., non-executive composite score; DSST, digit symbol substitution test; MoCA V-E., visual/executive subcomponent of MoCA; MoCA Abstr., abstraction subcomponent of MoCA; MoCA Mem., memory subcomponent of MoCA; MoCA Orien., orientation subcomponent of MoCA; QTcD, ECG QT dispersion–controlled for heart rate. Pearson correlation score for MoCA Orientation in the high QTcD group could not be calculated because everyone answered the question correctly, and the variable was constant. ^*a*^Whole sample; ^*b*^High QTcD sample; ^*c*^Low QTcD sample. *p < 0.05; ** p < 0.01.

In order to limit the role of physical fitness on the association between cardiac autonomic control (QTcD) and cognition, we limited our sample selection only to inactive older adults, as this was an inclusion criterion. We further tested if the aerobic fitness (VO_2_Peak) was correlated with QTcD, in order to decide if VO2 Peak can be included as a potential mediator. In order to test the hypothesis that QTcD predicts cognition, the primary analysis consisted of testing the association between QTcD and global MoCA within the low and high QTcD subgroups. Secondary exploratory analysis investigated if a specific pattern of cognitive functions emerges as being associated with QTcD. The cognitive variables used at this stage are the executive functions composite score, non-executive composite score, DSST, DTC, TSC, and the MoCA subscores.

## Results

**FIGURE 1:**
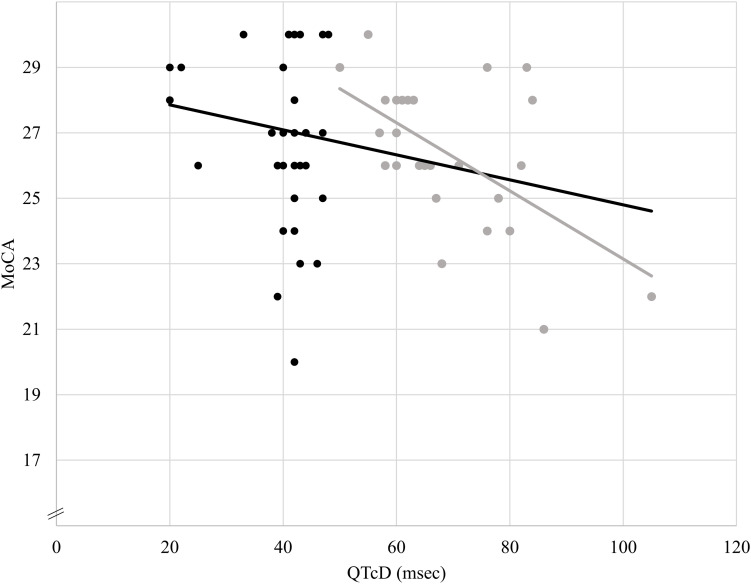
Association between QTcD and MoCA scores. The darker trend line shows the association between QTcD values and MoCA scores in the whole sample (*n* = 63, *R*^2^ = 0.17, β = –0.36). The lighter gray trend line shows the association between QTcD values and MoCA scores only in individuals with high QTcD values (QTcD > 47 msec, *n* = 28, *R*^2^ = 0.33, β = –0.58).

The primary outcome of this study consisted of the global MoCA score. Partitioning QTcD revealed almost an equal split between those in low (*n* = 34) and high (*n* = 29) groups. Table 1 provides demographic data. VO_2_Peak did not significantly correlate with QTcD (*p* > 0.05), and therefore could not be further investigated as mediator. Table 2 shows bivariate correlations between QTcD and all cognitive variables. Notable correlations found QTcD to correlate with global MoCA only in the whole sample (*r* = −0.28, *p* < 0.05) and in the high QTcD group (*r* = −0.58, *p* < 0.05). The primary analysis within the whole sample revealed that QTcD was a significant predictor of the global MoCA score (*F*_(4.58)_ = 3.00, *p* < 0.05, *R*^2^ = 0.17, β = −0.36, *p* < 0.01). It is important to note that within the high QTcD group, QTcD explained significantly more variance on the global MoCA score (*R*^2^ = 0.34, *F*_(4.23)_ = 2.99, *p* < 0.04, β = −0.57, *p* < 0.01; Table 3 and Figure 1), than in the whole sample. No such association was found to be significant in the low QTcD group (*p* > 0.05).

**TABLE 3:** Results of multiple linear regression models.

**Independent variable**	**Dependent Variable**	**β**	**SE**	***T***	***P*-value**
QTcD (whole sample)	Global MoCA	−0.36	0.02	−2.92	0.005
QTcD (high QTcD group)	Global MoCA	−0.57	0.03	−3.29	0.003
QTcD (high QTcD group)	Vis/Exec MoCA	−0.34	0.01	−1.90	0.070
QTcD (high QTcD group)	Language MoCA	−0.60	0.01	−3.60	0.002
QTcD (high QTcD group)	Abstraction MoCA	−0.43	0.01	−2.47	0.021
QTcD (high QTcD group)	Executive Composite Score	−0.45	0.01	−2.66	0.014
QTcD (high QTcD group)	DSST	−0.41	0.23	−2.35	0.028
QTcD (high QTcD group)	Dual Task–Task Set Cost	0.46	1.77	2.98	0.007

*DSST, Digit Symbol Substitution Test; MoCA, Montreal Cognitive Assessment; QTcD, ECG QT dispersion–controlled for heart rate; Vis/Exec MoCA, Visual/Executive subcomponent of MoCA; β is the standardized beta coefficient.*

Secondary exploratory analyses involved the MoCA subcomponents and the other cognitive scores recorded. All significant associations reported here were observed within the high QTcD group (Table 2). Analysis investigating the subcomponents of MoCA found QTcD to predict the language component (*R*^2^ = 0.39, *F*_(4.23)_ = 3.74, *p* < 0.05, β = −0.60, *p* < 0.01), and the abstraction component (*R*^2^ = 0.35, *F*_(4.23)_ = 3.04, *p* = 0.04, β = −0.43, *p* < 0.05; Table 3). Results also showed that the QTcD significantly predicted the executive composite score of the tablet tasks (*R*^2^ = 0.37, *F*_(4.23)_ = 3.38, *p* = 0.03, β = −0.45, *p* < 0.01), the digit symbol substitution score (*R*^2^ = 0.34, *F*_(4.23)_ = 2.93, *p* = 0.04, β = −0.41, *p* < 0.05), and the task set cost in the DT (*R*^2^ = 0.47, *F*_(4.23)_ = 5.13, *p* < 0.01, β = 0.46, *p* < 0.01; Table 3). No significant associations were found between QTcD and the secondary cognitive variables among individuals with low QTcD values.

## Discussion

The current study investigated the link between QTcD and cognition in healthy inactive older adults. The results show that higher QTcD values were associated with lower global and executive cognitive scores, and this relationship was even more apparent in individuals with high QTcD values. In other words, individuals having higher heterogeneity in ventricular repolarization exhibited lower cognitive functions. This study included an extensive cognitive assessment battery which allowed the identification of specific cognitive domains that are linked to ECG parameters. Among all of them, general executive functions, processing speed, and the ability to maintain multiple response alternatives in working memory were shown to be associated with QTcD. The findings using the median split show that some results are restricted to the high QTcD group, suggesting a discontinuous relationship between QTcD and cognition. The association between QTcD and global MoCA score was stronger in the high QTcD group relative to the whole sample (High QTcD *R*^2^ = 0.33 vs. all sample QTcD *R*^2^ = 0.17), and the other secondary cognitive variables were linked to QTcD only in the high QTcD group. This might suggest that only individuals with higher values in ventricular repolarization might show a stronger association with poorer cognitive abilities. This is in accordance with previous work suggesting that higher values on QTcD have more predictive value and are less likely to be affected by measurement error (Malik and Batchvarov, 2000).

Although the relationship between ventricular repolarization dispersion and cognition has been documented in dementia (Zulli et al., 2005; Coppola et al., 2013), to our knowledge, this is the first study to find a similar relationship in healthy individuals. This study further expands existing knowledge by identifying ventricular repolarization as an important marker of cognitive performance in healthy inactive older adults before the onset of a cardiovascular disease or clinical cognitive decline. The cognitive performance linked to QTcD in the present study included executive functions and processing speed. These results are consistent with previous findings that also identify executive functions (inhibition, updating and switching) and psychomotor speed as the cognitive functions most likely to be associated with a better autonomic cardiovascular regulation (measured by HRV) in a population-based sample (Stenfors et al., 2016). This is not trivial in a prevention perspective as executive functions have been shown to predict further global cognitive decline in prospective studies (Clark et al., 2012). Low executive functions were also shown to predict future functional decline and increased mortality (Johnson et al., 2007; Gross et al., 2016). As such, detecting subtle changes in executive functions with non-invasive ECG parameters could help identify those at greater risk of future cognitive decline and who need more aggressive preventive strategies.

One potential explanation for the link between QTcD and cognition in aging is that both the autonomic function and cognitive abilities degrade at the same time as a result of age-related deteriorations of the prefrontal cortex. Evidence suggests that both autonomic regulation and cognition (especially executive functions) in healthy older adults are controlled in part by the prefrontal cortex as part of the central autonomic network (Duschek et al., 2009; Thayer et al., 2009; Shah et al., 2011; Nonogaki et al., 2017). The neurovisceral integration model suggests that the cognitive abilities, autonomic function and emotional regulation are supported by a common hub of neural structures that includes the prefrontal cortex (Thayer and Lane, 2009). During normal aging, the frontal cortex is affected first, and it declines faster than other structures (Drag and Bieliauskas, 2010). As a result, it is possible that the functions controlled by this area are impaired, including executive functions and autonomic regulation. This top-down explanation is also supported by a recent study showing a reduction in the inter-connectedness between the autonomic system and cognition following stroke (Beer et al., 2017). In this population, the sudden brain damage was associated with lower cognitive abilities and a decreased parasympathetic activity compared to individuals without stroke. In addition, the autonomic activity of the stroke patients failed to show an association with cognitive functions, and it was less likely to adapt during a DT. Higher ventricular repolarization heterogeneity was also observed in older individuals immediately after stroke, and the prognosis after hospital discharge was poorer among individuals with higher variation in ventricular repolarization scores (Rahar et al., 2016).

The link observed between QTcD and cognition in aging could also reflect subtle cardiovascular declines that can impact both autonomic regulation and cognitive functioning. QTcD has been shown to be higher in individuals suffering from certain conditions known to be associated with lower cognitive functions, like hypertension (Malik and Batchvarov, 2000). Therefore, changes in ventricular repolarization could reflect subtle deteriorations of the cardiovascular system which can have a negative impact on brain health and eventually result in lower cognitive functions. For example, a recent study has showed that autonomic function markers predicts future white matter hyperintensities (Obara et al., 2018). In addition, the authors suggest that the white matter hyperintensities can be caused by the gradual deterioration of the cardiovascular system. As a result, it is possible that subtle, non-clinical cardiovascular declines could result in cardiac, autonomic and cognitive declines simultaneously. Similarly, other cardiovascular variables that were not measured as part of the present study should be considered to better document the relationship between cardiovascular health and cognition.

Although the use of QTcD as a clinical tool to predict the risk of mortality and sudden cardiac death has been challenged (Rautaharju et al., 2009), poor QTcD has still been linked to numerous cardiovascular conditions even beyond the increased risk of developing arrhythmia. For example, QTcD was found to be higher in individuals with hypertension, coronary disease, chronic myocardial infarction, left ventricular hypertrophy, heart failure, dilated cardiomyopathy, hypertrophic cardiomyopathy, acute myocardial infarction, and long QT syndrome (Malik and Batchvarov, 2000; Guntekin et al., 2011). In addition, a higher QTcD value was associated with an even higher severity of the disease (Malik and Batchvarov, 2000). The current results bring further evidence to the importance of high QTcD values as those were the ones to better predict cognitive abilities in this sample. Therefore, future longitudinal studies should investigate the predictive properties of QTcD as a potential indicator of future cognitive deficits, and its ability to detect people at risk of dementia.

The electrical functioning of the heart can be influenced by exercise, various cardiovascular problems (like hypertension and arrhythmias) as well as natural aging which are all known to remodel the heart over time (Devereux et al., 1983; Kang, 2006; Havmoller et al., 2007). In the present sample the participants were inactive, and had a relatively low VO_2_Peak indicating a low cardiovascular fitness. It is possible that the link between ventricular repolarization and cognition might be more evident in those individuals with low fitness or that are sedentary. Previous studies have indeed highlighted the link between sedentary behavior and lower cognitive abilities (Falck et al., 2017). Since the present sample was recruited to be very homogeneous in terms of physical fitness and physical activity, and since the VO_2_Peak values did not correlate with the variables of interest, there is not enough evidence to suggest that the association between QTcD and cognition observed in this study is mediated by physical fitness. On the other hand, future studies should investigate this in more detail by looking at the potential benefits of exercise training on cognitive abilities and autonomic regulation, and how this intervention might affect the link between QTcD and cognition. Indeed, it is already well documented that regular physical activity and exercise interventions show benefits on cognitive functioning, cardiac health, and autonomic regulation independently (Rosenwinkel et al., 2001; Dupuy et al., 2018; Ludyga et al., 2020). The benefit of exercise on cognition could be multidetermined, having the potential to improve autonomic regulation, cardiac functioning, and promote brain neuroplasticity, all of which can independently or synergistically result in improved cognitive abilities.

Overall, the results of this study agree with the current literature linking autonomic regulation and cardiac health to cognition. The present study is important because it offers novel insight into the heart-brain connection and reveal ventricular repolarization as an important ECG parameter associated with cognition in healthy older adults. Overall, the results show that higher heterogeneity in ventricular repolarization (QTcD) can be detected before the onset of any cardiovascular disease, and this is linked to lower cognitive abilities. Future studies should investigate this further by looking at the potential benefit of improved autonomic cardiovascular regulation on cognition through various interventions such as exercise.

## Data Availability Statement

The raw data supporting the conclusions of this article will be made available by the authors, upon reasonable request.

## Ethics Statement

This study involving human participants was reviewed and approved by the Research Ethics Board of the Geriatric Institute of Montreal (Le Comité d’Éthique de la Recherche Vieillissement-Neuroimagerie du CIUSSS du Centre-Sud-de-l’île-de-Montréal). The participants provided their written informed consent to participate in this study.

## Author Contributions

TV wrote the first draft of the manuscript, collected data, did the statistical analysis, and contributed to the main hypothesis. GL-L extracted and analyzed the ECG data and contributed to the main hypothesis. NK supervised the statistical analysis. AE supervised the study and collected data. TTV had clinical supervision of the participants included in the study and along with AN offered clinical feedback on the results interpretation. NB contributed to the development of the physical tests used in the study. LB contributed to the conception and design of the study, secured funding, and supervised the participant recruitment, testing, data collection, and data interpretation. All authors contributed to manuscript revision, read, and approved the submitted version.

## Conflict of Interest

The authors declare that the research was conducted in the absence of any commercial or financial relationships that could be construed as a potential conflict of interest.
